# DMA of TPU Films and the Modelling of Their Viscoelastic Properties for Noise Reduction in Jet Engines

**DOI:** 10.3390/polym14235285

**Published:** 2022-12-03

**Authors:** Moritz Neubauer, Michael Pohl, Michael Kucher, Robert Böhm, Klaus Höschler, Niels Modler

**Affiliations:** 1 Institute of Lightweight Engineering and Polymer Technology (ILK), Technische Universität Dresden, Hol-beinstraße 3, 01307 Dresden, Germany; 2 Chair of Aero Engine Design, Brandenburg University of Technology Cottbus-Senftenberg, Siemens-Halske-Ring 14, 03046 Cottbus, Germany; 3 Faculty of Engineering, Leipzig University of Applied Sciences, PF 30 11 66, 04251 Leipzig, Germany

**Keywords:** acoustic liner, dynamic mechanical analysis (DMA), fractional model, generalized Maxwell model, pre-smoothing, thermoplastic polyurethane (TPU), viscoelastic material properties

## Abstract

Due to current developments in jet engine design, the acoustic performance of conventional acoustic liners needs to be improved with respect to lower frequency spectrums and broadband absorption. In this context, the present study aimed to determine the viscoelastic material properties of a thermoplastic polyurethane (TPU) film for targeted application in novel acoustic liners with integrated film material for enhanced noise reduction. Therefore, a dynamic mechanical analysis (DMA) was performed to determine these viscoelastic material properties. Based on the acquired data, the time-temperature shift (TTS) was applied to obtain the material’s temperature- and frequency-dependent mechanical properties. In this regard, the William-Landel-Ferry (WLF) method and an alternative polynomial approach determining the shift factors were investigated and compared. Furthermore, a generalized Maxwell model—so-called Prony-series—with and without pre-smoothing utilizing of a fractional rheological model was applied to approximate the measured storage and loss modulus and to provide a material model that can be used in finite element analyses. Finally, the results were discussed concerning the application of the films in acoustic liners under the conditions of a standard flight cycle and the applied loads. The present investigations thus provide a method for characterizing polymer materials, approximating their mechanical behavior for vibration applications at different ambient temperatures and enabling the identification of their operational limits during the application in acoustic liners.

## 1. Introduction

Reducing aircraft engine noise has been a well-known challenge for a long time. The Certification Specification CS-36 of the European Union Aviation Safety Agency (EASA) regulates the permissible noise emissions that each engine of large aeroplanes must comply with [1]. Adapted designs will increase the engine’s bypass ratio (BPR) in the coming decades, e.g., geared turbofan engines, to comply with increasingly stringent future fuel consumption requirements [2]. Due to the higher BPR and the resulting increase in blade diameter, the fan speed must be reduced in order not to significantly exceed the speed of sound and to avoid aerodynamic losses due to compression shocks [3]. Therefore, the fan as the main noise contributor will decrease its rotational speed to limit the circumferential speed of the fan blade tips. Consequently, a lower rotational speed influences the tonal frequency directly. It shifts the audible frequency spectrum in a range of lower frequencies with a broadband character, making it even more challenging to find appropriate noise attenuation solutions [2].

In modern engines, acoustic-liners—in the form of honeycomb sandwich structures—are applied in the inlet and along the bypass duct. These rigid sandwich structures consist of a closed back plate, a perforated cover layer and honeycomb cells as a core [4]. The acoustic effect of a standard Helmholtz resonator liner is attenuated to a specific resonance frequency by its fixed geometry, leading to a narrowband character that is insufficient for future engine designs with higher BPR and lower broadband frequencies. The aforementioned specific acoustic liner is called a ‘Single Degree of Freedom-Liner’ (SDoF-Liner). The absorbed frequency of the Helmholtz effect correlates directly with the height of the cavities. Larger cell volumes are required to shift to lower frequencies [5]. This leads to conflicting targets, as the structure of the engine cowling has to be as thin and short as possible due to the strict requirements regarding the lightweight and aerodynamic design. Therefore, the available design space is strictly limited, and higher cores are not desirable. Nevertheless, placing a second liner over the first to damp the other, possibly lower frequency is another alternative. This ‘Double Degree of Freedom-Liner’ (DDoF-Liner) is separated by a second perforated layer between the two core structures [6].

Höschler et al. [7], Dannemann et al. [8], Knobloch et al. [9] and Mischke et al. introduced a new approach to acoustic liners with flexible wall structures. Honeycomb cells were replaced with a rectangular core pattern, and the rigid walls were partly substituted by flexible polymeric film walls that vibrate while acoustically excited. Due to the intrinsic material properties of the films, the vibrational energy is dissipated, and the noise emission is reduced. The first research results of Knobloch et al. [9] confirm the desired functionality. As a result of the film-forming ability of thermoplastic polyurethane (TPU) films [10] and their high damping ability [8], these materials are further analysed in the current study.

Investigations of the mechanical properties of polymers using dynamic mechanical analysis (DMA) can be readily found in the literature (compare [11,12]). Focusing particularly on the mechanical behavior of TPU, different authors analyzed the effect of additional components, such as talc [13], reduced graphene oxide and graphene nanoplatelets [14], long glass fiber reinforcement [15] or jute fiber [16]. Furthermore, using polymer TPU enables the fabrication of complex structures, such as lattice flexible TPU with honeycomb architecture [17], which can be characterized by DMA. The majority of these investigations concentrate on the thermal dependence of the mechanical properties. However, for acoustic applications in jet engines, both the influence of temperature and the excitation frequency must be determined and quantified. Thus, a complete description has to be given for typical operating temperatures and vibration frequencies in aviation.

This paper presents the material characterization of a TPU film for use in acoustic liners. A dynamic mechanical analysis with subsequent time-temperature shifting of the obtained measurement data was performed to determine the temperature- and frequency-dependent material properties. Furthermore, the Maxwell-Wichert model was applied to describe the viscoelastic behavior independent of the excitation speed of the film material. Based on the determined material properties, the application potential of the material in jet engines is discussed with regard to a standard flight cycle.

## 2. Materials and Methods

### 2.1. Material

**Figure 1:**
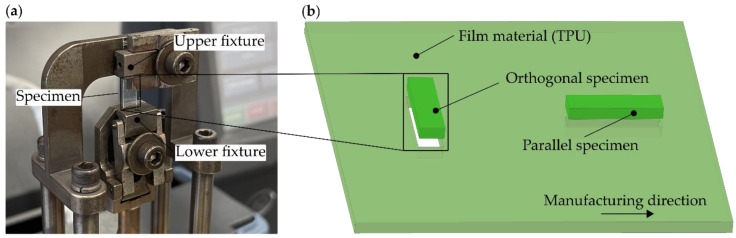
(**a**) Experimental setup of the DMA test in tensile mode, (**b**) schematic representation of the orthogonal and parallel removal of the film specimen with respect to the manufacturing direction of the film material.

The selected material is thermoplastic polyurethane (TPU), which refers to the thermoplastic elastomers (TPE) group. The polyether-based TPU (ELASTOLLAN 1170 A, BASF SE, Ludwigshafen, Germany) was selected because, in previous studies on a novel acoustic liner (see, e.g., [8,18]), TPU film (due to its damping properties) resulted in improved sound absorption compared to conventional liner structures. The TPU material was manufactured by Gerlinger Industries GmbH in a continuous melt extrusion process with a nominal width of 300 mm and a nominal thickness of 0.3 mm. In order to evaluate the possible changes in mechanical properties caused by the manufacturing process, the specimens were taken at 0° (parallel) and 90° (orthogonal) to the manufacturing direction of the film. All specimens were taken from the film web’s center and had average dimensions of 14 mm × 4 mm × 0.3 mm. The experimental setup of the DMA and a schematic view of the specimen preparation is shown in Figure 1.

### 2.2. Experimental Apparatus and Procedure 

The dynamic mechanical analysis was conducted on a Q800 DMA (TA Instruments, Eschborn, Germany) in compliance with DIN EN ISO 6721. This measurement equipment was used to determine the material’s complex modulus [11]

(1) $$E^{*}=E^{\prime }+jE^{\prime \prime }$$

where $E^{\prime }$ was the storage modulus and $E^{\prime \prime }$ was the loss modulus. The loss factor was defined by the ratio $\tan\;\delta =E^{\prime \prime }/E^{\prime }$. All quantities were measured over the temperature range from -60°C to 100°C in 10 K steps at the frequencies of f = 0.1, 0.3, 0.5, 1, 2, 3, 5, 10, 30, 50 Hz. The applied static load was 0.5 N and the oscillating amplitude 20 µm.

The time-temperature shift (TTS) was used to further evaluate the determined DMA values to define the temperature-dependent mechanical properties of the viscoelastic material. Thus, it is possible to extrapolate $E^{\prime }$, $E^{\prime \prime },$ and tan δ depending on the load frequency or the load duration over longer examination periods. At the same time, it was possible to determine the glass transition temperature $T_{g}$. The master curve was created for $T_{ref}=20{}^{\circ}C$ and the average values of the specimens were examined. The description of the shift factors of the TTS for further application in finite element analysis (FEA) was carried out using the William-Landel-Ferry (WLF) shift function [19].

(2) $$\log\left(a_{T}\right)=\frac{-C_{1}\cdot \left(T-T_{ref}\right)}{\left|C_{2}+\left(T-T_{ref}\right)\right|}$$

where T refers to the temperature at which the material was examined, $T_{ref}$ is the reference temperature, $C_{1}$ and $C_{2}$ are characteristic material constants and $a_{T}$ the shift factor for all recorded temperature measurements. In addition, an alternative characterization approach used in previous studies, such as [20,21], was implemented, allowing an approximation based on an eighth-degree polynomial function:

(3) $$\log\left(a_{T}\right)=\overset{k=8}{\underset{i=0}{\sum }}a_{i}T^{i}.$$

### 2.3. Viscoelastic Material Modelling

**Figure 2:**
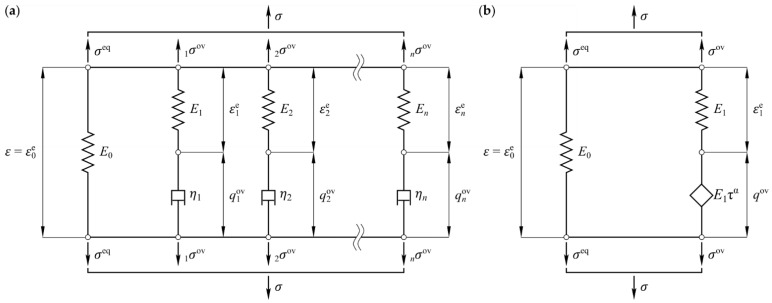
Schematic representation of used rheological models: (**a**) generalized Maxwell model, (**b**) fractional four-parameter model.

The material behavior of the TPU films depends on the deformation rate and the ambient temperature. For small deformations, this behavior can be described by means of the linear viscoelasticity theory (compare, e.g., [22]). The generalized Maxwell model (MM) is a standard model to characterize the viscoelastic behavior. This model consists of an elastic spring element with Young’s modulus $E_{0}$ and the parallel connection of several Maxwell elements (Figure 2a). The Maxwell elements each consist of an additional elastic spring element with the moduli $E_{i}$ and a viscous damper with the viscosity $\eta_{i}$. Therefore, the relaxation time is the ratio $\tau_{i}=\eta_{i}/E_{i}$. The superposition of n Maxwell elements leads to a discrete relaxation spectrum, which is also known as Prony-series. From this, the real $E^{߰}$ and the imagery part $E^{߰߰}$ of the complex modulus $E^{*}$ are defined by (compare, e.g., [23])

(4) $$E^{\prime }\left(\omega \right)=E_{0}+\overset{n}{\underset{i=1}{\sum }}E_{i}\frac{\omega^{2}\tau_{i}^{2}}{1+\omega^{2}\tau_{i}^{2}},$$

(5) $$E^{\prime \prime }\left(\omega \right)=\overset{n}{\underset{i=1}{\sum }}E_{i}\frac{\omega \tau_{i}}{1+\omega^{2}\tau_{i}^{2}}$$

where ω=2πf is the angular frequency. An alternative viscoelasticity model can be formulated using a fractional element [24,25]

(6) $$\sigma^{\mathrm{ov}}=E_{1}\tau^{\alpha }D^{\alpha }q^{\mathrm{ov}}$$

where the stress is proportional to the fractional derivative of the strain $q^{\mathrm{ov}}$, and $D^{\alpha }$ is the Riemann–Liouville definition of the fractional derivative [26]. The parallel connection of the elastic spring element with Young’s modulus $E_{0}$ and a series connection consisting of an additional elastic spring with the modulus $E_{1}$ and a fractional element according to Equation (6). This model represents a fractional four-parameter model with the relaxation time τ and the order of fractional calculus α (Figure 2b). The stress-strain relation of this fractional model (FM) can be combined to

(7) $$D^{\alpha }\sigma +\frac{1}{\tau^{\alpha }}\sigma =\left(E_{0}+E_{1}\right)D^{\alpha }\varepsilon +\frac{E_{0}}{\tau^{\alpha }}\varepsilon$$

Using the Fourier transforms of the fractional-order operator [27]

(8) $$ℱ\left[D^{\alpha }f\left(t\right)\right]={\left(j\omega \right)}^{\alpha }\tilde{f}\left(\omega \right)$$

the stress-strain relation Equation (8) can be written in the frequency domain as

(9) $${\left(j\omega \right)}^{\alpha }\tilde{\sigma }\left(\omega \right)+\frac{1}{\tau^{\alpha }}\tilde{\sigma }\left(\omega \right)=\left(E_{0}+E_{1}\right){\left(j\omega \right)}^{\alpha }\tilde{\varepsilon }\left(\omega \right)+\frac{E_{0}}{\tau^{\alpha }}\tilde{\varepsilon }\left(\omega \right)$$

where $\tilde{\sigma }\left(\omega \right)$ is the Fourier transform of the stress and $\tilde{\varepsilon }\left(\omega \right)$ is the Fourier transform of the strain. Rearranging Equation (9) yields the storage modulus $E^{\prime }$ and the loss modulus $E^{\prime \prime }$ of the four-parameter model

(10) $$E^{\prime }\left(\omega \right)=E_{0}+\frac{E_{1}{\left(\omega \tau \right)}^{\alpha }\left[\cos\left(\frac{\alpha \pi }{2}\right)+{\left(\omega \tau \right)}^{\alpha }\;\right]}{1+2{\left(\omega \tau \right)}^{\alpha }\cos\left(\frac{\alpha \pi }{2}\right)+{\left(\omega \tau \right)}^{2\alpha }},$$

(11) $$E^{\prime \prime }\left(\omega \right)=\frac{E_{1}{\left(\omega \tau \right)}^{\alpha }\sin\left(\frac{\alpha \pi }{2}\right)}{1+2{\left(\omega \tau \right)}^{\alpha }\cos\left(\frac{\alpha \pi }{2}\right)+{\left(\omega \tau \right)}^{2\alpha }}$$

The generalized Maxwell model (Prony-series), according to Equations (4) and (5), is used in the following to approximate the measured mechanical behavior of TPU. Here, the approximation is made without and with smoothing—so-called pre-smoothing—of the measured data to reduce the waviness carried out using the fractional four-parameter model as defined in Equations (10) and (11). A number of n Maxwell elements were used to quantify the mechanical behavior for the frequency range under consideration. Additionally, one Maxwell element was added one frequency decade below, and one element was added one frequency decade above the considered frequency range. This results in a total number of Maxwell elements of n+2.

For the identification process of the parameter $E_{0}$, $E_{i}$, $\tau_{i}$ with i=1,2,…,n, Park and Kim [28] demonstrated an approach for fitting the Prony-series on the basis of data from relaxation tests. For this data set, they used a power-law pre-smoothing followed by the fitting of the Prony-series due to local irregularities of the measured data. For the fitting of the Prony-series, the relaxation times were selected so that the time range was equally divided on a logarithmic scale (compare [28]). In the current study, the approach of Park and Kim was adapted to the data obtained using DMA. Therefore, the relaxation times $\tau_{i}=1/f_{i}$ were equally divided on a logarithmic scale; the distance between the individual relaxation times depends on the total number of Maxwell elements n. Therefore, the considered frequency range starts one frequency decade below and one frequency decade above the measured frequency range. In this study, the approximation ability of the Maxwell model was investigated for a number of n≥3 Maxwell elements. By quantifying the resulting error between experiment and model, the approximation ability of the model was evaluated. The corresponding moduli $E_{i}$ were determined utilizing a non-linear least square fit approach. In this method, a weighted sum of squares, $h\left(\vec{p}\right)$, of the differences between the experimental, $E_{k}^{\prime }$ and $E_{k}^{\prime \prime }$, and the model, $E_{k,\mathrm{calc}}^{\prime }$ and $E_{k,\;\mathrm{calc}}^{\prime \prime }$ modulus is minimized by choosing the best values of the adjustable parameters $\vec{p}$ and minimizing the weighted differences between the experimental and calculated model moduli:

(12) $$h\left(\vec{p}\right)∶=\overset{N}{\underset{k=1}{\sum }}\left\{w_{k}^{\prime }{\left[E_{k}^{\prime }-E_{k,\mathrm{calc}}^{\prime }\right]}^{2}+w_{k}^{\prime \prime }{\left[E_{k}^{\prime \prime }-E_{k,\mathrm{calc}}^{\prime \prime }\right]}^{2}\right\},$$

where N is the number of all calculated frequencies. In the case of previous pre-smoothing of measured data, $E_{k}^{\prime }$ and $E_{k}^{\prime \prime }$ are the calculated values of the fractional four- parameter model’s approximation.

In the current study, proportional weighting $w_{k}^{\prime }=1/{\left(E_{k}^{\prime }\right)}^{2}$ and $w_{k}^{\prime \prime }=1/{\left(E_{k}^{\prime \prime }\right)}^{2}$ of Equation (12) was used. As a measure of the relative error of the approximated complex modulus, the following equation was used:

(13) $$r^{2}\approx \frac{1}{2N}\overset{N}{\underset{k=1}{\sum }}\left\{{\left[\frac{E_{k}^{\prime }-E_{k,\mathrm{calc}}^{\prime }}{E_{k,\mathrm{calc}}^{\prime }}\right]}^{2}+{\left[\frac{E_{k}^{\prime \prime }-E_{k,\mathrm{calc}}^{\prime \prime }}{E_{k,\mathrm{calc}}^{\prime \prime }}\right]}^{2}\right\}$$

Using this error measure in Equation (13), the different introduced rheological models were assessed with regard to their approximation ability.

## 3. Results and Discussion

### 3.1. Analysys of Production-Related Material Orthotropy

**Figure 3:**
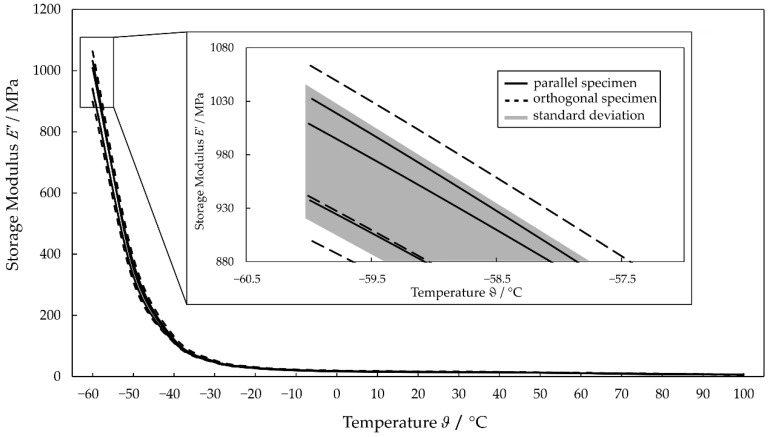
DMA results for the specimen in the parallel and orthogonal directions.

The evaluation of the DMA specimens taken parallel and orthogonal with respect to the production direction shows that the values of $E^{\prime }$ and $E^{\prime \prime }$ vary considerably for both directions. In this context, at a frequency of 50 Hz, the orthogonal specimens have the highest and the lowest $E^{\prime }$ values of all the measured specimens (see Figure 3). The curves of the parallel specimen are randomly interposed. Thus, no distinct direction-dependent material behavior can be observed concerning the parallel and orthogonal specimens.

For a more detailed evaluation of the acquired data, the variations of the measured values at 50 Hz for all six specimens are analyzed (see Table 1). The largest absolute variations of $E^{\prime }$ are to be expected at the lowest measured temperature of −60 °C, since $E^{\prime }$ increases significantly with decreasing temperature. The absolute difference between the lowest and highest measured values is 163 MPa for the orthogonal specimens 4 and 5. In contrast, the measured variations of $E^{\prime }$ for the parallel specimens are much smaller, with a maximum of 95 MPa. The absolute deviation at a temperature lower than −30 °C, where the transition from glass to the rubbery state causes significant changes in the physical properties, increases for all specimens (compare the glass transition temperature ($T_{g}$) of TPUs [29]). The relative variations of the values around the mean value are 5.5% up to approximately 70 °C. At higher temperatures and lower $E^{\prime }$, the measurement inaccuracy of the test equipment has a more significant effect, resulting in higher standard deviations of over 11% at 100 °C.

**Figure 4:**
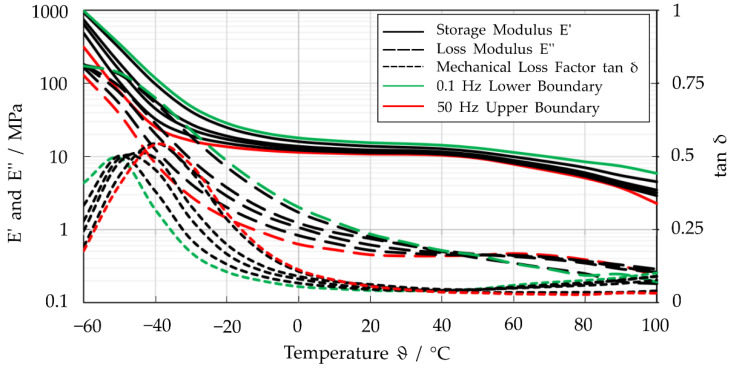
Averaged values for storage modulus $E^{\prime }$, loss modulus $E^{\prime \prime }$ and mechanical loss factor tanδ at the frequencies of f = 0.1, 0.3, 0.5, 1, 2, 3, 5, 10, 30, 50 Hz.

Since no significant direction-dependent material behavior was observed, no distinction was made between orthogonal and parallel specimens in the following investigations. The $E^{\prime }$ or $E^{\prime \prime }$ curves, representing the averaged data of all specimens for the investigated frequency range as a function of temperature, are shown in Figure 4.

The results show that the storage modulus increases with decreasing frequencies (see Figure 4). A similar behavior is observed for the loss modulus between −60 °C and approximately 45 °C. However, for higher temperatures, the highest and lowest $E^{\prime \prime }$ switch from 1 Hz and 50 Hz. The course of the curves allows the glass transition temperature to be set to −53 °C.

### 3.2. Frequency Dependent Complex Modulus

**Figure 5:**
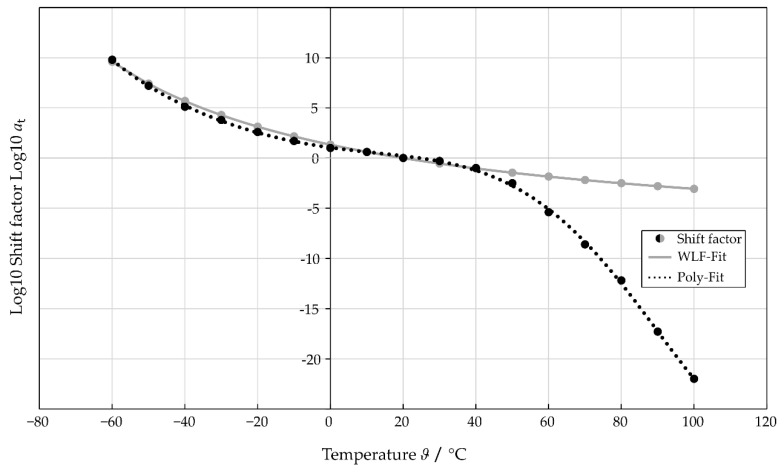
Calculated shift factor as a function of temperature based on the WLF method and an eighth-degree polynomial approximation.

The shift factors were first determined using the WLF method (see Equation (2)) to perform the TTS. In this context, the constants $C_{1}$ = 9 and $C_{2}$ = 155 were derived from the measurement results by curve fitting. In addition, an approximation for the material behavior over the entire temperature range was determined using an eighth-degree polynomial approximation and compared to the WLF shift factors (see Figure 5). Both approximations have a similar course for the temperature range from $T_{g}$ to $T_{Ref}$. However, at about 40 °C, the WLF method deviates strongly from the polynomial approximation. This is in accordance with the stated range of validity of the WLF method of about 100 °C above $T_{g}$ [19]. Consequently, an eighth-degree polynomial approximation can give a more accurate description of the shift curve progression at higher temperatures.

**Figure 6:**
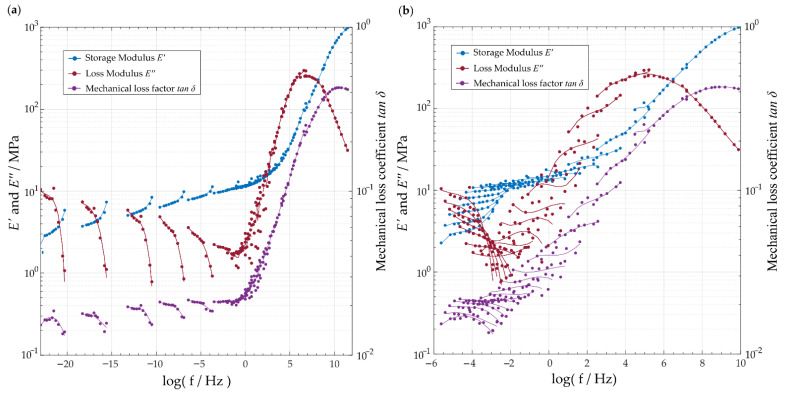
Master curves generated by shifting $E^{\prime }$ and $E^{\prime \prime }$ using the TTS principle with respect to a reference temperature of 20 °C: (**a**) using an eighth-degree polynomial approximation and (**b**) the WLF shift factors.

In order to display and discuss the resulting differences, the master curves with a reference temperature of 20 °C were created for both methods (see Figure 6a,b). In this context, the horizontal data shift of $E^{\prime }$, $E^{\prime \prime }$ and tan δ was performed using a logarithmic scale of the frequency axis. The individual data sets were represented by a trend line in the form of a polynomial approximation to visualize the course of the different data sets of the DMA (see Figure 6a,b).

The master curve generated from the polynomial approach (see Figure 6a) shows a discontinuous approximation of the correlation for the value range lower than log(f/Hz)=4. However, for the value range above log(f/Hz)=4, the master curve provides a smooth course that approximates the material behavior in the designated frequency range. In contrast, the master curve generated with the shift factors of the WLF method shows a comparatively discontinuous approximation to the material behavior for the same value range, exhibiting a greater deviation from the fitting curves in the individual data sets (see Figure 6b). Consequently, for a more accurate description of the frequency-dependent material behavior of TPU, the master curve generated from the polynomial approximation seems to be more feasible and thus was used in the following.

### 3.3. Identified Viscoelastic Material Parameter

**Figure 7:**
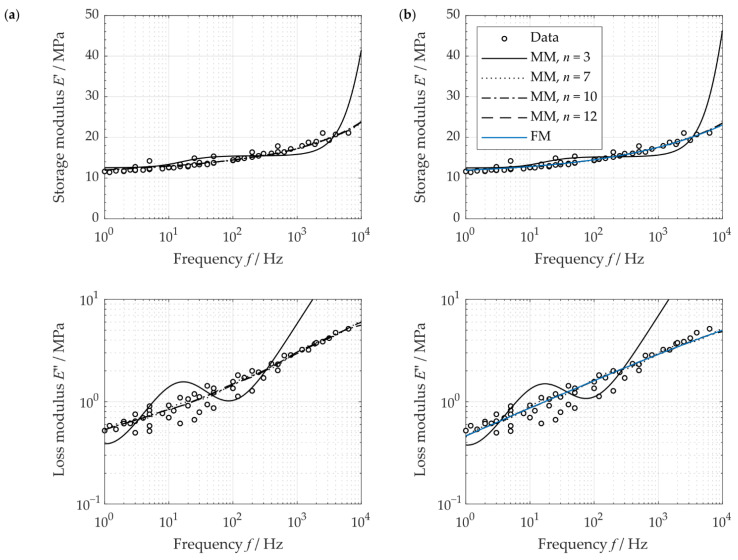
Approximated storage and loss modulus using generalized Maxwell models and a fractional model: (**a**) Prony series without pre-smoothing, (**b**) Prony series with pre-smoothing.

For the application case of a jet engine, the frequency range of interest is from 5 Hz to values below 2000 Hz (compare, e.g., RTCA DO-160G). Considering the nearest frequency decades, this leads to the considered frequency range from 1 Hz to 10,000 Hz (compare Figure 7).

**Figure 8:**
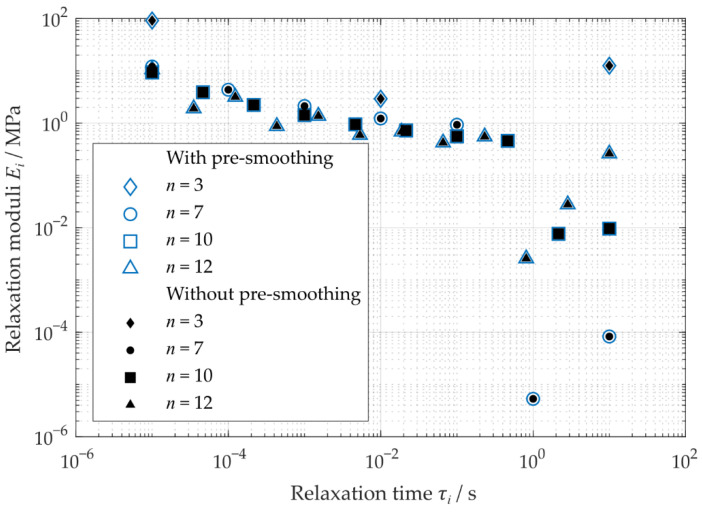
Resulting relaxation moduli of Prony-series of the generalized Maxwell model without and with pre-smoothing.

It can be seen that the approximation ability increased with an increasing number of Maxwell elements. Furthermore, the resulting error measure decreased (Table 2). The relaxation moduli obtained similar results for the different number of Maxwell elements n. Only the values between a relaxation time of τ=1 s and τ=10 s show high deviations (Figure 8).

Here, the pre-smoothing of the data shows no considerable influence on the resulting relaxation moduli and overall relative errors of the determined Prony-series. Due to this pre-smoothing, the courses determined here tend to show a slight increase in the resulting error. For this reason, a pre-smoothing of the measured data does not improve the approximation of the moduli and thus should not be used.

Considering the resulting error, the error only slightly decreases for the number of Maxwell elements of more than n=7. This represents at least one or more Maxwell element(s) per frequency decade. Kim and Park [28] and Chae et al. [20] also used one Maxwell element per times decade or frequency decade in their study. Thus, in the following, a number of n=7 was chosen to estimate the material’s time-dependent behavior.

The generalized Maxwell model enables the description of the frequency-dependent material behavior of the investigated TPU films, considering linear viscoelastic deformations. Furthermore, this model is implemented in conventional FE software.

### 3.4. Effect of Flight Cycle Conditions on Mechanical Properties

**Figure 9:**
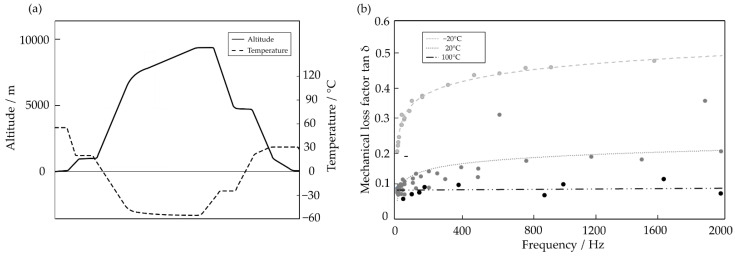
(**a**) Schematic flight cycle with a typical temperature curve; (**b**) Change of the mechanical loss factor in dependency of standard sinusoidal vibration test’s excitation frequency (compare RTCA DO-160G).

Based on the experimental results of the DMA and the analysis of the TTS, it is evident that the viscoelastic material properties of the TPU film vary significantly within the analyzed frequency and temperature range. Therefore, it is essential to evaluate the material properties of the applied film material of an acoustic liner with regard to the prevailing environmental conditions of a flight cycle considering temperature and vibration (see Figure 9). The acoustic liner is exposed to a wide temperature range during operation. However, the critical points for compliance with the legal standards are limited to specific points during the flight path. For admission, it is necessary to comply with the International Civil Aviation Organization (ICAO, Annex 16, Chapter 14). In this context, the lateral full-load reference, the overflight reference and the approach reference are particularly important in reducing sound levels due to their proximity to ground level. With regard to the potential temperature scenarios, assuming a temperature range of −20 to 50 °C within the targeted flight sections seems reasonable (Figure 9a). At the same time, the different conditions of taxiing, taking-off, climbing, approaching and landing cause different vibration conditions inside the engine. The curve of a typical test procedure for airborne equipment of a fixed-wing aircraft with turbofan engines in analogy to the RTCA DO-160G varies between 5 Hz and 2000 Hz, as mentioned above. The different vibration scenarios, which depend on the excitation frequency and the acceleration amplitudes, have a direct effect on the material’s viscoelastic behavior and thus on the system’s acoustic performance. Investigations by Kucher et al. [30] show that thermoplastic polymers, especially at high frequencies, lead to self-heating under sinusoidal excitation. For this reason, it can be assumed that the mechanical properties are in a complex relationship with the existing ambient, load and geometric conditions and must be analyzed for the respective application on the basis of experimental tests.

Since the mechanical loss coefficient is an important material parameter concerning the design of acoustic liners, as Neubauer et al. [18] demonstrated, its frequency dependence is determined for the specified temperatures of −20 °C, 20 °C and 100 °C (Figure 9b). The temperature dependence resulted in a significant difference between the individual damping curves. High temperatures reduce the difference between the damping values at 5 Hz and 2000 Hz.

Consequently, a material with high damping properties is required for acoustic liners in jet engines. These materials should have a minimal dependency on temperature and frequency. However, concerning the large temperature and frequency range during a flight cycle, changes in material properties are unavoidable, especially for polymers. Thus, the frequency and temperature dependency need to be considered in the design process of acoustic liners. In this context, narrowing the value range for frequency and temperature, targeting only the crucial phases of flight, could lead to a more suitable material selection and improve the acoustic liner’s overall performance.

The introduced experimental approach is only applicable to homogeneous materials and the description of linear viscoelastic behavior due to the requirements of the used temperature time superposition. These materials were defined as thermorheologically simple polymeric materials (compare, e.g., [31]). In the presented investigations, the frequency- and temperature-dependent viscoelastic material properties of a thermoplastic film for use in novel acoustic liners were determined and analyzed with respect to the real conditions of a jet engine.

## 4. Conclusions

The results of the dynamic mechanical analysis of the thermoplastic polyurethane film showed no distinct direction-dependent material behavior as a result of the manufacturing process. In addition, concerning the temperature-dependent course of the viscoelastic properties, the established WLF method and a polynomial approach were evaluated for the calculation of the shift factors. For the time-temperature shift, the polynomial approach was preferred, which shows a more consistent and accurate approximation of the material behavior. The resulting frequency-dependent viscoelastic properties (storage modulus, loss modulus) were modelled employing the Prony-series to obtain a feasible material model commonly used in finite element analyses. Finally, the viscoelastic properties of the film were evaluated with respect to the targeted application of acoustic liners in jet engines, showing the dependence of the material parameters on temperature and frequency. Therefore, they should be considered in the design process of acoustic liners, especially in the material selection phase.

## Figures and Tables

**Table 1:** Variation of the experimental storage modulus $E^{\prime }$ at 50 Hz.

Temperature [°C]	$E^{\prime }\;\left[MPa\right]$						
	Specimen 1 Parallel	Specimen 2 Parallel	Specimen 3P arallel	Specimen 4 Orthogonal	Specimen 5 Orthogonal	Specimen 6 Orthogonal	Relative Standard Deviation [%]
−60	1032.22	1009.03	937.32	1063.32	899.23	941.61	5.94
−40	118.79	121.74	112.63	131.49	111.11	109.92	6.37
−20	28.10	29.06	27.22	31.07	28.11	26.83	4.90
0	17.17	18.40	16.76	19.75	17.96	17.17	5.61
20	14.41	15.79	14.24	17.07	15.55	15.08	6.17
40	13.68	14.40	13.54	15.28	14.23	14.19	3.97
60	10.81	11.44	10.70	12.30	11.41	11.33	4.61
80	6.98	8.93	8.26	9.43	8.51	8.41	8.92
100	0.00	6.44	5.98	6.55	5.56	4.74	11.25

**Table 2:** Resulting error measure of the approximated storage and loss modulus.

n	3	7	10	12
*Without pre-smoothing*				
Error measure r	0.294	0.111	0.109	0.109
*With pre-smoothing*				
Error measure r	0.298	0.124	0.122	0.122

## Data Availability

The data presented in the current study are available on request from the corresponding author.
